# ECT‐AD: Baseline Demographics of a Novel Therapeutic Intervention for Severe Agitation and Aggression in Dementia

**DOI:** 10.1002/alz.085641

**Published:** 2025-01-09

**Authors:** Brent P. Forester, Julia G. Merrill, Adriana P. Hermida, Maria I. Lapid, Martina Mueller, Louis J. Nykamp, Regan E. Patrick, Jefferson Mattingly, Steve J. Seiner, Georgios Petrides

**Affiliations:** ^1^ Tufts Medical Center, Boston, MA USA; ^2^ McLean Hospital, Belmont, MA USA; ^3^ Emory University, Atlanta, GA USA; ^4^ Mayo Clinic, Rochester, MN USA; ^5^ Medical University of South Carolina, Charleston, SC USA; ^6^ Pine Rest Christian Mental Health Center, Grand Rapids, MI USA; ^7^ Harvard Medical School, Boston, MA USA; ^8^ RWJ Barnabas Health, West Orange, NJ USA; ^9^ Northwell Health, Glen Oaks, NY USA

## Abstract

**Background:**

Effect and Safety of Electroconvulsive Therapy plus Usual Care for the Acute Management of Severe Agitation in Dementia (ECT‐AD) is a multi‐site NIA‐funded FDA‐regulated pioneering clinical trial to investigate the effectiveness of electroconvulsive therapy (ECT) in treating severe and treatment‐refractory agitation and aggression among individuals with advanced dementia, a condition that has a profound negative impact on patient quality of life and caregiver burden. Here we present baseline demographics of the patient population in this ongoing trial.

**Method:**

To date we have enrolled 18 participants, with a mean age of 74.1 years, where majority are male (61.1%). The racial composition is predominantly White (94.4%), with Asian representation (5.6%, 1/18), and with 11.1% (2/18) identifying as Hispanic or Latino. Dementia subtypes in our cohort include Alzheimer’s disease (AD, 77.8%), vascular dementia (VaD, 16.7%, 3/18), and frontotemporal dementia (FTD, 5.6%, 1/18).

**Result:**

Baseline assessments reveal severe cognitive and functional impairment, as indicated by a mean Mini‐Mental State Examination (MMSE) score of 4.0, Barthel Index (BI) score of 52.2, and SIB‐8 score of 2.9, all consistent with advanced stages of dementia. High mean total scores on the Cohen‐Mansfield Agitation Inventory (CMAI, 75.8), Neuropsychiatric Inventory (NPI) agitation (15.1) and aggression (9.2) scales, and Pittsburgh Agitation Scale (PAS, 7.8), reflect the severe levels of agitation and aggression in our participants.

**Conclusion:**

These baseline demographic and clinical data underscore the profound impact of advanced dementia, highlighting the need for innovative treatment approaches like ECT. The ECT‐AD trial offers a critical step towards understanding and improving the management of agitation in dementia, with implications that could enhance current clinical care practices.

